# Emergency abdominal surgery after solid organ transplantation: a systematic review

**DOI:** 10.1186/s13017-016-0101-6

**Published:** 2016-08-30

**Authors:** Nicola de’Angelis, Francesco Esposito, Riccardo Memeo, Vincenzo Lizzi, Aleix Martìnez-Pérez, Filippo Landi, Pietro Genova, Fausto Catena, Francesco Brunetti, Daniel Azoulay

**Affiliations:** 1 Department of Digestive, Hepatobiliary Surgery and Liver Transplantation, “Henri Mondor” University Hospital, Université Paris Est - UPEC, 51, Avenue du Maréchal de Lattre de Tassigny, 94010 Créteil, France; 2 Department of Hepato-biliary and Pancreatic Surgery, Nouvel Hôpital Civil, Strasbourg, France; 3 Department of Emergency Surgery, University Hospital “Ospedale Maggiore” of Parma, Parma, Italy

**Keywords:** Emergency abdominal surgery, General surgery, Solid organ transplantation, Liver transplantation, Kidney transplantation, Heart transplantation, Lung transplantation, Systematic review

## Abstract

**Aims:**

Due to the increasing number of solid organs transplantations, emergency abdominal surgery in transplanted patients is becoming a relevant challenge for the general surgeon. The aim of this systematic review of the literature is to analyze morbidity and mortality of emergency abdominal surgery performed in transplanted patients for graft-unrelated surgical problems.

**Methods:**

The literature search was performed on online databases with the time limit 1990–2015. Studies describing all types of emergency abdominal surgery in solid organ transplanted patients were retrieved for evaluation.

**Results:**

Thirty-nine case series published between 1996 and 2015 met the inclusion criteria and were selected for the systematic review. Overall, they included 71671 transplanted patients, of which 1761 (2.5 %) underwent emergency abdominal surgery. The transplanted organs were the heart in 65.8 % of patients, the lung in 22.1 %, the kidney in 9.5 %, and the liver in 2.6 %. The mean patients’ age at the time of the emergency abdominal surgery was 49.4 ± 7.4 years, and the median time from transplantation to emergency surgery was 2.4 years (range 0.1–20). Indications for emergency abdominal surgery were: gallbladder diseases (80.3 %), gastrointestinal perforations (9.2 %), complicated diverticulitis (6.2 %), small bowel obstructions (2 %), and appendicitis (2 %). The overall mortality was 5.5 % (range 0–17.5 %). The morbidity rate varied from 13.6 % for gallbladder diseases to 32.7 % for complicated diverticulitis. Most of the time, the immunosuppressive therapy was maintained unmodified postoperatively.

**Conclusions:**

Emergency abdominal surgery in transplanted patients is not a rare event. Although associated with relevant mortality and morbidity, a prompt and appropriate surgery can lead to satisfactory results if performed taking into account the patient’s immunosuppression therapy and hemodynamic stability.

## Background

Organ transplantation is considered as the most effective treatment for end-stage disease of the heart, lung, pancreas, liver, and kidney, with approximately 28000 solid organs transplanted every year in Europe and USA, and overall 114690 organs transplanted in 2012 worldwide [1]. The high number of transplantations per year and the long-term graft survival had contributed to drastically increase the likelihood for an emergency surgeon to encounter a transplanted patient with a graft-unrelated surgical problem [2–5].

The management of graft-unrelated acute abdominal disease in transplanted patients generally adheres to the fundamental principles of any surgical treatment. Preoperative evaluation should consider that transplanted patients are chronically immunosuppressed, and although most of them achieve an excellent functional capacity and are able to live normal productive lives, they remain at increased risk for any surgical complication, particularly infectious. Moreover, the clinical presentation of many disease may be different from the general population, sometimes leading to misdiagnosis or underestimation of the disease severity [5]. Another important issue is the potential impact of any operative procedure on the functional capacity of the transplanted organ, which shows to have a reduced clinical reserve compared to the native organ. Even modest intra-operative insults, such as hypotension, may negatively affect the transplanted organ, and thus, when several options are available, the most cautious, conservative, minimally invasive, and standardized surgical approach should be preferred in these patients [5].

When considering the incidence of several common general surgical problems and the ever-larger cohort of transplanted patients living and functioning under chronic immunosuppression [6], it becomes apparent that all general surgeons, especially outside of transplant centers, should be familiar with the factors that influence the surgical outcomes in this particular subset of patients, along with the issues that are likely to affect the optimal surgery timing and the postoperative cares [6].

The aim of the present systematic review is to provide an exhaustive analysis of the current available literature about the outcomes of emergency abdominal surgery (EAS) performed in transplanted patients for graft-unrelated abdominal diseases. The evidence-based assessment of EAS morbidity and mortality in transplanted patients may help the surgeon and clinician in the decision making process face to the challenging management of acute abdominal disease in this particular subset of patients.

## Materials and methods

The methodological approach included the development of selection criteria, definition of search strategies, assessment of study quality, and abstraction of relevant data. The PRISMA statements checklist for reporting a systematic review was followed [7].

### Study inclusion criteria

The study selection criteria were defined before initiating data collection for proper identification of studies eligible for the analysis. All studies in which the primary objective was to describe EAS for graft-unrelated diseases in transplanted patients were retrieved and analyzed.

### Types of study

Epidemiological studies, interventional trials, case–control studies, cross-sectional studies and case series including more than four patients [8] were considered eligible for inclusion in this systematic review. Case reports, review articles, systematic reviews, meta-analyses, conference abstracts, letters and commentaries were not considered.

### Types of participants

Patients who had received a solid organ transplantation (heart, lung, liver, pancreas, or kidney) presenting with graft-unrelated surgical abdominal diseases were considered.

### Types of intervention

All types of surgical abdominal emergencies (e.g. appendectomy, cholecystectomy, colectomy, bowel resection, gastric resection, surgical repair of incisional hernia, explorative laparotomy) were considered.

### Types of outcome measures

The primary outcomes were the post-operative 90-day morbidity and mortality following EAS. All secondary parameters (e.g. hospital stay and immunosuppressive therapy) reported in the selected studies were also evaluated.

### Literature search strategy

A literature search was performed on the following online databases: MEDLINE (through PubMed), EMBASE, Scopus, Cochrane Oral Health Group Specialized Register, and ProQuest Dissertations and Thesis Database. To increase the probability of identifying all relevant articles, a specific research equation was formulated for each database, using the following keywords and/or MeSH terms: emergency, emergency surgery, urgent surgery, appendicitis, diverticulitis, perforation, cholecystectomy, colectomy, appendectomy, cholecystitis, humans, adult, transplant, transplantation, solid organ transplantation, transplant patient, transplanted patient.

In addition, the reference lists from the eligible studies and relevant review articles (not included in the systematic review) were crosschecked to identify additional records. The literature search was performed on January 2016 and was restricted to articles published since 1990. Only studies written in English and meeting the selection criteria were reviewed.

### Study selection and quality assessment

The titles and abstracts of the retrieved studies were independently and blindly screened for relevance by two reviewers (FE and VL). To enhance sensitivity, records were removed only if both reviewers excluded the record at the title screening level. All disagreements were resolved by discussion with a third and fourth reviewers (NdeA and RM). Subsequently, both reviewers performed a full-text analysis of the selected articles. Two reviewers independently assessed the risk of bias and study quality by using appropriate tools. Precisely, The NICE guidelines [9] was used for the quality assessment of case series, which was rated on a 8 points scale by answering eight questions concerning the following aspects:, study setting (i.e. uni or multicentric), study hypothesis/objective, case definition, outcome definition, data collection, patient recruitment, result description and analysis. Additionally, the Grading of Recommendations Assessment Development and Evaluation (GRADE) system [10] was used to enable consistent judgment of the “body of evidence” included in the systematic review. GRADE specifies four categories: high, moderate, low, and very low. In the context of a systematic review, the quality of evidence reflects the confidence that the estimates of the effect are correct and overpasses the individual study risk of bias by evaluating the following aspects: study design, imprecision, inconsistency, indirectness of study results, and publication bias.

### Data extraction

Data extracted from the studies included in the systematic review were processed for qualitative and possibly quantitative analyses. Outcome measures (mean and median values, standard deviation, and ranges) were extracted for each variable. Average morbidity and mortality rates were calculated.

## Results

### Literature search and selection

**Fig. 1:**
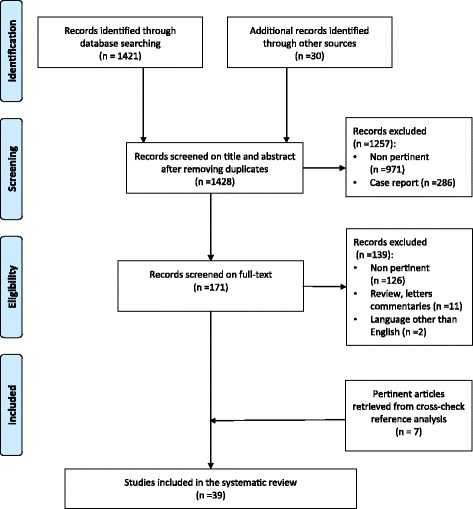
Flow chart of the study search, selection, and inclusion. Example of search equation: emergency[Title/Abstract]) OR emergency surgery[Title/Abstract]) OR urgent surgery[Title/Abstract]) OR appendicitis[Title/Abstract]) OR diverticulitis[Title/Abstract]) OR perforation[Title/Abstract]) OR cholecystectomy[Title/Abstract]) OR colectomy[Title/Abstract]) OR appendectomy[Title/Abstract]) OR cholecystitis[Title/Abstract]) AND Humans[Mesh] AND English[lang] AND adult[MeSH]) AND (((((transplant[Title/Abstract]) OR transplantation[Title/Abstract]) OR solid organ transplantation[Title/Abstract]) OR transplant patient[Title/Abstract]) OR transplanted patient[Title/Abstract])

Out of the 1428 articles initially identified, 39 articles [2, 4, 11–47] met the inclusion criteria and were selected for the systematic review. The flow chart of studies identification and inclusion/exclusion process is shown in Fig. 1.

### Study characteristics

The selected studies were published between 1996 and 2015 and they were all case series [2, 4, 11–47]. They were carried out in ten different countries, particularly in Europe (*n* = 11) [13, 14, 18, 24, 28–30, 34, 35, 38, 40], North America (*n* = 24) [2, 4, 11, 12, 15–17, 19, 21–23, 25–27, 31, 33, 36, 37, 39, 41, 43–46], Asia and Pacific (*n* = 4) [20, 32, 42, 47]. The overall number of transplanted patients considered was 71671. Of these patients, 1761 (2.5 %) underwent EAS for graft-unrelated acute diseases. The mean age of the patients undergoing EAS was 49.4 ± 7.4 years, and the median time from transplantation to the required emergency operation was 2.4 years (range 0.1–20). The organ transplanted was the heart in 65.8 % of cases, the lung in 22.1 %, the kidney in 9.5 % and the liver in 2.6 %. EAS was necessary because of the following conditions: gallbladder diseases (80.3 %), gastrointestinal perforations (9.2 %), complicated diverticulitis (6.2 %), small bowel obstruction (2 %), appendicitis (2 %), and miscellaneous (0.3 %). The overall mortality rate was 5.5 %.

The studies concerning gallbladder diseases [4, 11–23] requiring EAS in transplanted patients are displayed in Table 1. Acute cholecystitis, with and without gallstones (including hydrops, empyema, gangrene and perforation), was the most common primary diagnosis, followed by gallstones without cholecystitis, and chronic cholecystitis. Laparoscopic cholecystectomy was performed in 72 % of EAS cases and laparotomy in 23.1 %. In 1.9 % of patients a surgical cholecystostomy was carried out. In 42 patients, the type of surgery performed was not precisely described. The morbidity rate was 13.6 %. The most frequent EAS-related complications included: respiratory failure, pneumonia, deep venous thrombosis, pulmonary embolism, postoperative haemorrhage, and surgical site infection. The overall mortality was 3.4 %. The median hospital stay was 9.3 days (range 1–38).

**Table 1:** Reports of gallbladder diseases requiring EAS after solid organ transplantation

	Transplanted organ (n)				EAS characteristics and outcomes				
Authors, Year	Heart	Lung	Heart-Lung	Kidney	Patients undergoing EAS (n)	EAS Rate (%)	Surgical procedures (n)	Morbidity [n(%)]	Mortality [n(%)]
Taghavi S et al., 2015 [11]		9186			258	2.81	OC (58), LC (190), n/a (10)	48 (18.6)	0 (0)
Kilic A et al., 2013 [12]	23854				1054	4.42	OC (233), LC (795), CS (26)	126 (12.0)	37 (3.5)
Lahon B et al., 2011 [13]		351			11	3.13	OC (11)	n/a	1 (9.1)
Paul S et al., 2009 [4]		208			13	6.25	OC (4), LC (9)	n/a	0 (0)
Sarkio S et al., 2007 [14]				1608	17	1.06	n/a (17)	n/a	1 (5.9)
Takeyama H et al., 2006 [15]	402				2	0.50	n/a (2)	n/a	0 (0)
Englesbe MJ et al., 2005 [16]	168				7	4.17	LC (7)	2 (28.6)	1 (14.3)
Richardson WS et al., 2003 [17]	518				19	3.67	OC (4), LC (15)	9 (47.4)	1 (5.3)
Hoekstra HJ et al., 2001 [18]		125			1	0.80	LC (1)	n/a	0 (0)
Gupta D et al., 2000 [19]	143	30	5		6	3.37	OC (6)	3 (50)	3 (50)
Lord RV et al., 1998 [20]	455	133	57		13	2.02	n/a (13)	4 (30.8)	1 (7.7)
Bhatia DS et al., 1997 [21]	349				5	1.43	OC (5)	n/a	2 (40)
Milas M et al., 1996 [22]	175				5	2,86	OC (5)	0 (0)	0 (0)
Sharma S et al., 1996 [23]	240				2	0.83	OC (1), CS (1)	0 (0)	1 (50)
Total	26304	10033	62	1608	1413	3.72	OC (327), LC (1017), CS (27), n/a (42)	192 (13.6)	48 (3.4)

*EAS* emergency abdominal surgery, *OC* open cholecystectomy, *LC* laparoscopic cholecystectomy, *CS* cholecystostomy, *n/a* not available

The studies concerning gastrointestinal perforations requiring EAS in transplanted patients [4, 13, 18, 21, 23–36] are shown in Table 2. The most frequent causes of perforation were: diverticulitis (this disorder and its complications are described below and in Table 3); peptic disease; ischemia; chronic inflammatory bowel disease; iatrogenic factors; post-transplantation lymphoproliferative disorders; enteritis and colitis caused by Clostridium difficile or Cytomegalovirus. In immunosuppressed transplanted patients, the signs and symptoms of perforation were often absent or non-specific. Therefore, the interval from clinical onset to surgery was very large, ranging from 2 to 8 days. The diagnosis of perforation was confirmed by an abdominal and pelvic computed tomography (CT) scan in most of the cases, whereas abdominal X-rays were sufficient in some cases.

**Table 2:** Reports of gastrointestinal perforations requiring EAS after solid organ transplantation

	Transplanted organ (n)				EAS characteristics and outcomes				
Authors, Year	Liver	Heart	Lung	Kidney	Patients undergoing EAS (n)	EAS Rate (%)	Surgical procedures (n)	Morbidity [n (%)]	Mortality [n (%)]
Timrott K et al., 2014 [24]		342	1074		21^a^	1,48	BR (21)	n/a	n/a
Lee JT et al., 2014 [25]				2406	10	0.42	CR (10)	6 (60)	3 (30)
Cruz RJ Jr et al., 2012 [26]	5677				6	0.11	n/a (6)	n/a	2 (33.3)
Boutros M et al., 2012 [27]				814	2	0.25	CR (2)	n/a	0 (0)
Boutros M et al., 2012 [27]	430				3	0.70	CR (3)	n/a	1 (33.3)
Jorgensen KK et al., 2012 [28]	69				4	5.80	CR (4)	n/a	0 (0)
Lahon B et al., 2011 [13]			351		2	0.57	CR (2)	n/a	0 (0)
Paul S et al., 2009 [4]			208		8	3.85	n/a^c^ (7), CR (1)	n/a	3 (37.5)
Catena F et al., 2008 [29]				1611	46	2.86	CR (21), BR (15), UC (10)	n/a	11 (23.9)
Ho GT et al., 2005 [30]	413				4	0.97	CR (4)	n/a	0 (0)
Keven K et al., 2004 [31]				702	2	0.28	CR (2)	1 (50)	1 (50)
Karakayali H et al., 2002 [32]				1038	6	0.58	UC (4), CR (2)	n/a	0 (0)
Hoekstra HJ et al., 2001 [18]			125		2	1.60	BR (1), CR (1)	n/a	0 (0)
Andreoni KA et al., 1999 [33]				1417	26	1.83	CR (26)	n/a	1 (3,8)
Mueller XM et al., 1999 [34]		93			2^b^	2.15	UC (1), CS (1)	0 (0)	0 (0)
Bhatia DS et al., 1997 [21]		349			7	2.01	CR (7)	3 (42.9)	0 (0)
Wekerle T et al., 1997 [35]			124		4	3.23	n/a^c^ (2), CR (2)	n/a	4 (100)
Beaver TM et al., 1996 [36]			60		3	5.00	HP (3)	0 (0)	1 (33,3)
Sharma S et al., 1996 [23]		240			2	0.83	n/a^c^ (2)	0 (0)	1 (50)
Total	6589	1024	1942	7988	160	0.91	BR (37), CR (87), HP (3), UC (15), CS (1), n/a (17)	n/a	28 (17.5)

*EAS* emergency abdominal surgery, *BR* bowel resection, *CR* colon resection, *UC* ulcer closure, *CS* colostomy, *HP* hartmann procedure, *n/a* not available

^a^3out of 21 were after heart transplantation; ^b^1 Duodenal ulcer perforation and 1 iatrogenic colon perforation; ^c^Peptic ulcer disease

**Table 3:** Reports of complicated diverticulitis after solid organ transplantation

	Transplanted organ (n)					EAS characteristics and outcomes				
Authors, Year	Liver	Heart	Lung	Heart-Lung	Kidney	Patients undergoing EAS (n)	EAS Rate (%)	Surgical procedures (n)	Morbidity [n (%)]	Mortality [n (%)]
Larson ES et al., 2014 [37]			314			7	2.23	HP (7)	n/a	1 (14.3)
Scotti A et al., 2014 [38]					717	9	1.26	HP (9)	5 (55.6)	0 (0)
Reshef A et al., 2012 [39]		5329^a^				9	0.17	CR (9)	5 (55.6)	2 (22.2)
Reshef A et al., 2012 [39]					5329^a^	12	0.23	CR (12)	6 (50)	0 (0)
Reshef A et al., 2012 [39]	5329^a^					4	0.08	CR (4)	3 (75)	1 (25)
Reshef A et al., 2012 [39]			5329^a^			12	0.23	CR (12)	9 (75)	4 (33.3)
Paul S et al., 2009 [4]			208			2	0.96	CR (2)	n/a	2 (100)
Dalla Valle R et al., 2005 [40]					875	8	0.91	HP (5), S (2), n/a (1)	6 (75)	1 (12.5)
Miller CB et al., 2006 [41]			229			3	1.31	HP (3)	n/a	0 (0)
Goldberg JH et al., 2006 [2]		530	435	47		11	1.09	HP (11)	n/a	1 (9.1)
Qasabian RA et al., 2004 [42]		639	248	66		9	0.94	HP (6), S (1), S + DLI (2)	0 (0)	1 (11.1)
Karakayali H et al., 2002 [32]					1038	2	0.19	CR (2)	n/a	0 (0)
Hoekstra HJ et al., 2001 [18]			125			5	4	HP (3), S (2)	n/a	0 (0)
Khan S et al., 2001 [43]		233	35			2	0.75	HP (2)	1 (50)	0 (0)
Lederman ED et al., 1998 [44]					1137	13	1.14	HP (13)	1 (7.7)	1 (7.7)
Sharma S et al., 1996 [23]		240				2	0.83	S (2)	n/a	1 (50)
Total	5329^a^	1642	1594	113	3767	110	0.88	HP (59), CR (41), S + DLI (2), S (7), n/a (1)	36 (32.7)	15 (13.6)

*EAS* emergency abdominal surgery, *HP* hartman procedure, *S* Sigmoidectomy, *S + DLI* sigmoidectomy + diverting loop ileostomy, *CR* colonic resection, *n/a* not available

^a^All transplant patients poled together

The perforation was located at the level of colon in 58.4 % of patients, small bowel (including jejunum and ileum) in 33.8 %, stomach and duodenum in 7.8 %. All operations were performed by open approach. The surgical procedures carried out were: colon resection with primary anastomosis in 54.3 % of cases; small bowel resection with anastomosis in 23.2 %; stomach and duodenum ulcer closure in 9.4 %; Hartmann’s procedure in 1.9 %; and colostomy in 0.6 %. In 10.6 % of cases, the type of surgery performed could not be traced back. Generally, the immunosuppressive therapy was maintained unmodified postoperatively. The median hospital stay was 22.2 days (range 9–87), with an overall mortality rate of 17.5 %.

The studies specifically dealing with complicated diverticulitis [2, 4, 18, 23, 32, 37–44] occurring in transplanted patients and requiring EAS are displayed in Table 3. Among the most frequent clinical manifestations, there were fever, abdominal pain, signs of localized or diffuse peritonitis, anorexia, diarrhea and leukocytosis. Abdominal and pelvic CT scan was performed for all patients, showing complicated diverticulitis, including free perforations, phlegmons and abscesses. The surgical approach was laparotomy in all cases. It consisted in: Hartman’s procedure in 53.6 % of patients; colon resection with primary anastomosis in 37.3 %; sigmoidectomy in 6.4 %; and sigmoidectomy with diverting loop ileostomy in 1.8 %. In 0.9 % of cases the type of surgery performed was not clearly reported. The immunosuppressive therapy was maintained unmodified postoperatively. The morbidity rate was 32.7 %, with the most frequent complications being severe respiratory diseases and wound infection. The overall mortality rate was 13.6 %, in most cases due to sepsis. No case of acute transplant rejection was reported.

The studies concerning small bowel obstructions occurring in transplanted patients and requiring EAS [4, 13, 21, 26, 32, 33, 41] are shown in Table 4. The most frequent causes of small bowel obstruction were post-transplantation lymphoproliferative disorders and mechanical obstruction due to intestinal adhesions. Abdominal pain, chronic diarrhea and lower gastrointestinal bleeding were the most common clinical signs and symptoms. Abdominal-pelvic CT scan was performed in most of the patients. In some cases, the diagnosis was based on abdominal X-rays. Surgery consisted in exploratory laparotomy, with small bowel resection in 57.1 % of cases and lysis of adhesions in 42.9 %. The immunosuppressive therapy was maintained post-operatively or reduced in some patients. The overall mortality rate was 14.3 %, mostly because of sepsis or as a direct consequence of surgical complications.

**Table 4:** Reports of small bowel obstructions requiring EAS after solid organ transplantation

	Transplanted organ (n)				EAS characteristics and outcomes				
Authors, Year	Liver	Heart	Lung	Kidney	Patients undergoing EAS (n)	EAS Rate (%)	Surgical procedures (n)	Morbidity [n (%)]	Mortality [n (%)]
Cruz RJ Jr et al., 2012 [26]	5677				7	0,12	BR (7)	n/a	2 (28.6)
Lahon B et al., 2011 [13]			351		3	0.85	BR (3)	n/a	0 (0)
Paul S et al., 2009 [4]			208		14	6.73	LA (14)	n/a	1 (7.1)
Miller CB et al., 2006 [41]			229		2	0.87	BR (1), LA (1)	n/a	1 (50)
Karakayali H et al., 2002 [32]				1038	1	0.10	BR (1)	n/a	0 (0)
Andreoni KA et al., 1999 [33]				1417	4	0.28	BR (4)	n/a	1 (25)
Bhatia DS et al., 1997 [21]		349			5	1.43	BR (5)	1 (20)	0
Total	5677	349	788	2455	36	0.39	BR (21), LA (15)	n/a	5 (13.9)

*EAS* emergency abdominal surgery, *BR* bowel resection, *LA* lysis of adhesions *n/a* not available

The studies dealing with appendicitis in transplanted patients requiring EAS [18, 23, 24, 32, 41, 45, 46] are displayed in Table 5. Abdominal pain was present in 95.5 % of patients, with associated nausea, vomiting, fever, diarrhea, and leukocytosis. Physical examination demonstrated right lower quadrant tenderness in 90.1 % of the patients. Abdominal and pelvic CT scan was performed in all cases and showed signs of acute appendicitis, including a non-filling appendix, appendicolith, pericecal stranding, or free fluid. Furthermore, the 36.3 % of patients underwent ultrasound examination revealing appendicitis.

**Table 5:** Reports of appendicitis requiring EAS after solid organ transplantation

	Transplanted Organ (n)						EAS characteristics and outcomes			
Authors, Year	Liver	Heart	Lung	Kidney	Pancreas	Small Bowel	Patients undergoing EAS (n)	EAS Rate %	Morbidity [n (%)]	Mortality [n (%)]
Timrott K et al., 2014 [24]		342					2	0.58	n/a	n/a
Miller CB et al., 2006 [41]			229				2	0.87	n/a	0 (0)
Abt PL et al., 2005 [45]	n/a						7	n/a	2 (28.6)	0 (0)
Savar A et al., 2005 [46]	3287	1336	231	3053	6	10	17	0.21	4 (23.5)	0 (0)
Karakayali H et al., 2002 [32]				1038			5	0.48	n/a	0 (0)
Hoekstra HJ et al., 2001 [18]			125				2	1.6	n/a	0 (0)
Sharma S et al., 1996 [23]		240					1	0.42	n/a	0 (0)
Total	3287^a^	1918	585	4091	6	10	36	0.29^a^	n/a	0 (0)

^a^excluding Abt PL et al.’s study for lack of the number of recipients; *EAS* emergency abdominal surgery, *n/a* not available

The time from abdominal pain onset to appendectomy varied from 14 h to 4 days. Pathologic examination demonstrated appendicitis in 81.8 % of cases. Appendicular perforation occurred in 22.7 % of patients, more frequently in those ones operated belatedly. Negative appendectomy was observed in four specimens (18.2 %). The levels of calcineurin inhibitors were titrated in the post-operative period and maintained at pre-operative values. Patients receiving steroids or mycophenolate resumed their pre-operative dosing immediately after surgery. All patients received a minimum of 24 h intravenous antibiotic treatment, with longer duration for patients with intraperitoneal contamination. The median duration of hospitalization was 6.3 days (range 1–20). No mortality was reported.

Other disorders necessitating EAS in transplanted patients included complicated incisional hernia, pancreatic abscess, and splenic infarction. These studies [23, 47] are shown in Table 6.

**Table 6:** Reports of EAS for various indications after solid organ transplantation

	Transplanted organ (n)		EAS characteristics and outcomes					
Authors, Year	Liver	Heart	Indication for EAS	Patients undergoing EAS (n)	EAS Rate (%)	Surgical procedures (n)	Morbidity [n (%)]	Mortality [n (%)]
Ozgor D et al., 2014 [47]	173		IH	3	1.73	HRP (3)	1 (33.3)	0 (0)
Sharma S et al., 1996 [23]		240	PA	2	0.83	DD (2)	n/a	0 (0)
Sharma S et al., 1996 [23]		240	SI	1	0.42	SP (1)	n/a	0 (0)
Total	173	240		6	1.45	HRP (3), DD (2), SP (1)	n/a	0 (0)

*EAS* emergency abdominal surgery, *IH* incisional hernia, *HRP* hernia repair with polypropylene mesh, *PA* pancreatic abscess, *DD* debridement/drainage, *SI* splenic infarction, *SP* splenectomy, *n/a* not available

### Study quality assessment

**Fig. 2:**
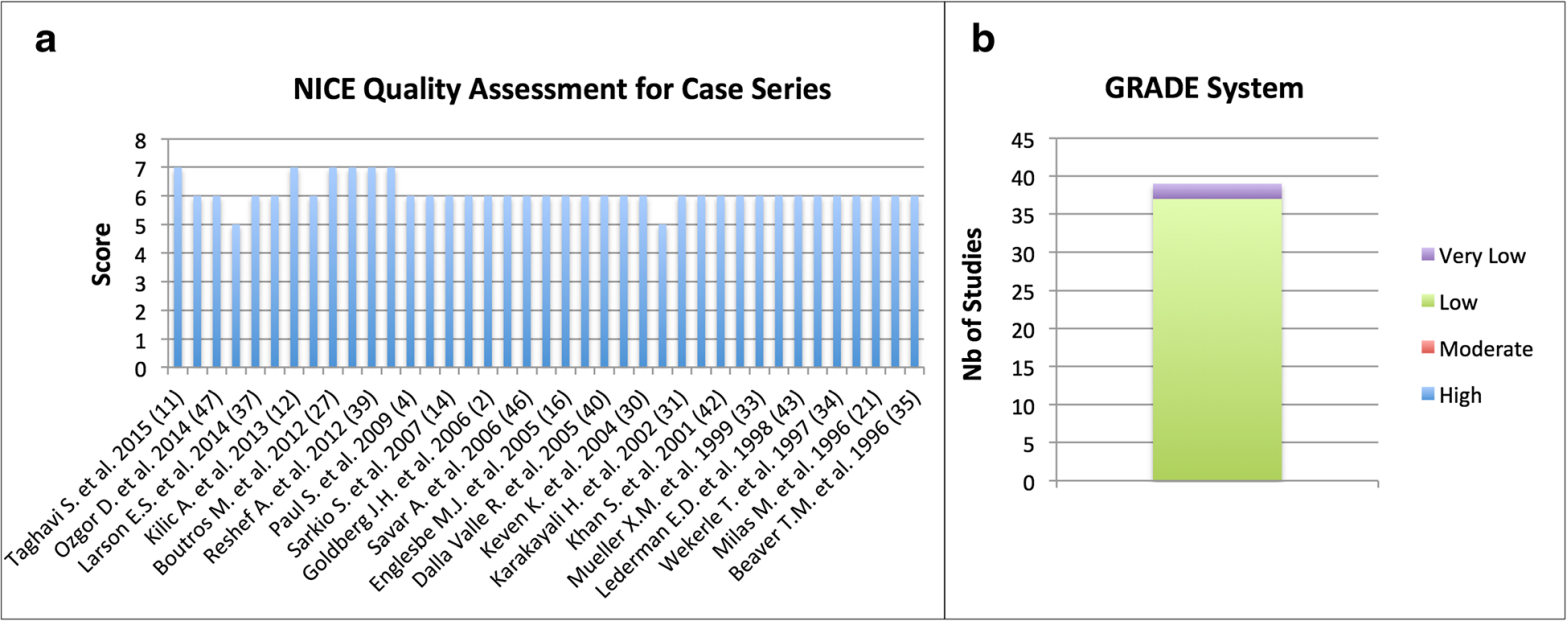
Study quality assessment by using the NICE (**a**) and GRADE (**b**) systems

Based on the NICE guidelines for the quality assessment of case series [9], 6 studies received a score of 7/8 [11–13, 27, 28, 39], 31 studies were graded 6/8 [2, 4, 14–16, 18–23, 25, 26, 29–38, 40–47] and 2 studies 5/8 [17, 24]. Based on the GRADE system [48], 37 studies [2, 4, 11–16, 18–23, 25–47] (94.9 %) were judged as being of low quality, and the remaining 2 studies [17, 24] of very low quality of evidence. Of note, all studies were retrospective, which, by definition, are susceptible of major selection bias as well as misclassification or information bias due to the unknown accuracy of record keeping. NICE guidelines and GRADE system quality assessment are displayed in Fig. 2.

## Discussion

The present systematic review is the first to analyze the available literature concerning EAS in transplanted patients. Emergency surgery after transplantation for graft-unrelated acute diseases involved 2.5 % of patients, with the main causes being gallbladder diseases, gastrointestinal perforations, complicated diverticulitis, small bowel obstructions, and appendicitis. Overall, EAS was associated with high morbidity (up to 32.7 %) and mortality (up to 17.5 %) rates, which highlight the particularly challenging surgical management of transplanted patients.

Undoubtedly, one of the most important factors contributing to the high morbidity and mortality in transplanted patients operated for EAS is represented by the use of several immunosuppressive regimens, most frequently consisting in a triple-drug association: calcineurin inhibitors, antiproliferative agents, and corticosteriods [49]. In general, immunosuppressive therapy predisposes transplanted patients to various gastrointestinal diseases [50], lymphoproliferative disorders, infective complications (e.g. Cytomegalovirus, Clostridium difficile), and can mask the presenting signs and symptoms of many disease processes. Moreover, immunosuppression is known to interfere with the patient management for transplant-unrelated surgical procedures in terms of risk for drug interactions, adverse effects, wound healing, and postoperative complications [49].

Gallbladder diseases were the most frequent reason for EAS after transplantation, which was mainly reported in heart-transplanted patients. Despite actual controversies, the high incidence of biliary tract diseases may be related to cyclosporine-induced perturbation of bile composition resulting in an increased prevalence of biliary stones formation [51, 52]. Other putative explanations include vagotomy and associated biliary stasis, rapid weight loss following transplantation, atherosclerosis, and hemolysis [53]. EAS for gallbladder diseases has been associated with high morbidity (up to 47 %) [17, 20] and mortality rates (up to 29 %) [17, 19], and thus a prophylactic cholecystectomy in asymptomatic patients awaiting transplantation has been proposed as a strategy to avoid symptomatic gallstone diseases later on [54, 55]. This may be particularly indicated before heart and lung transplantation, after which a high incidence of clinical manifestations and increased mortality has been reported [19, 56, 57]. However, this approach remains under debate and it is not routinely performed, also because an emergency cholecystectomy can be highly problematic in patients with end-stage diseases [58]. Both laparoscopy and laparotomy were reported for the surgical management of emergency related to gallbladder diseases. Some evidence suggests that patients undergoing open cholecystectomy develop major post-operative complications (Dindo-Clavien Classification [59] >3) more frequently than patients operated on by laparoscopy [11]. Other studies showed that laparoscopic cholecystectomy can be performed safely in lung-, and kidney-transplanted patients [11, 60], whereas pancreas-transplanted patients may require specific technical modifications in the laparoscopic cholecystectomy procedure, which need to be carefully evaluated preoperatively. In the present systematic review it was not possible to evaluate the rate of post-operative complications and mortality following EAS for gallbladder diseases by specific surgical approach. Nevertheless, the overall morbidity and mortality rates appears to be higher than those found in the literature for non-transplanted patients (estimated at <1 %) [61–64].

Gastrointestinal perforations were the second most frequent cause of EAS after solid organ transplantation. The majority (57.5 %) of the described cases in the literature occurred in kidney-transplanted patients for polycystic kidney disease. Although no precise etiology was found, it seems that transplanted patients for polycystic kidney disease develop more gastrointestinal complications after transplantation than kidney-transplanted patients for other diseases [33], probably due to several biologically active substances that influence the alimentary tract and contribute to the increased incidence of ileus in these patients [65].

In particular, complicated diverticulitis requiring EAS in transplanted patients showed an incidence rate of 0.88 %, which is in accordance with other studies on transplanted patients (1–4 %) [2, 66, 67] and definitely higher than in the general population (estimated incidence of 0.025–0.053 %) [42, 68, 69]. The diagnosis and treatment of diverticulitis after solid organ transplantation are challenging, since immunosuppressive therapies may mask presenting signs and symptoms and impair the ability to contain the infective process [70]. Often, the clinical manifestation and physical examination do not reflect the severity of intra-abdominal disease; signs of infection, such as fever and tachycardia, especially in heart-transplanted patients, could be absent or highly attenuated. Abdominal examination and laboratory testing may be irrelevant, and only the abdominal CT scan appears to be a reliable diagnostic tool to determine the location and severity of the disease. Moreover, morbidity and mortality following emergency colectomy for complicated diverticulitis in transplanted patients are higher (32.7 and 13.6 %, respectively) than those observed in immunocompetent individuals [29, 39].

Another gastrointestinal complication observed in transplanted patients was small bowel obstruction. The most frequent etiologies were post-transplant lymphoproliferative disorders and mechanical obstruction due to adhesions. The first disease is usually diagnosed within the first 2 years after transplantation, and it is strongly associated with high levels of immunosuppressive drugs [71]. The concomitant involvement of both small and large bowels may occur in one third of the patients [72]. These findings stressed the importance of early recognition and systematic referral for endoscopy of any transplanted patient with gastrointestinal symptoms, particularly over the first 2 years after transplantation [26].

Finally, EAS for appendicitis in transplanted patients was described in 0.29 % of cases, mainly in liver-transplanted patients (38.9 %). Appendicitis is one of the most common surgical disease in the general population, with an estimated lifetime risk of 8.6 % in males and 6.7 % in females [73], however only few studies are found in transplanted patients. In all case series, appendectomy was approached by laparotomy. Only a case report in the literature describes laparoscopic appendectomy in two liver and renal transplanted patients with excellent results similar to those in non-transplanted patients [74]. It must be noted that appendicitis in transplanted patients is frequently associated with delayed diagnosis or misdiagnosis, which can lead to complicated appendicitis including rupture and gangrene. As a general rule, appendicitis should be aggressively treated to minimize morbidity in the clinical setting of chronic immunosuppression. Based on the literature findings, surgical morbidity and mortality rates associated with this emergency procedure appear to be much lower than for other gastrointestinal complications [18].

In almost all the included studies, immunosuppressive regimens were maintained unchanged during the post-operative period after EAS. Although the heterogeneity of the literature does not allow pooling data together and analyzing the direct impact of immunosuppressive therapies on post-operative complications, the role of immunosuppression remains crucial since it claims for a more aggressive treatment of acute abdominal diseases. On the other hand, this may contribute to have no cases of graft failure or late rejection following EAS.

### Study limitations

The currently available literature on EAS in transplanted patients for graft-unrelated abdominal diseases is based on case series only, which represents a low quality of evidence. However, it may not be feasible to perform randomized controlled trials or even case–control studies in the setting of emergency surgery. Moreover, it was not possible in the present systematic review to perform any quantitative syntheses or risk analysis due to the observational nature of the included studies, the high heterogeneity, and the lack of control groups. Thus, caution should be paid in the interpretation of the results since several biases can be mentioned in the individual studies, such as selection bias, reporting bias, publication bias, and geographical bias. However, we tried to control for search biases by searching the literature on multiple databases, by manual crosscheck of the reference lists, and by performing the critical appraisal by two independent reviewers.

## Conclusion

Given the growing number of transplantations per year and the long-term graft survival, EAS for graft-unrelated acute diseases in transplanted patients is not a rare event. The risk of misdiagnosis or delayed diagnosis in chronically immunosuppressed transplanted patients should be minimized by an attentive evaluation of all clinical signs and symptoms. Whenever surgery is indicated, a prompt surgical approach can achieve satisfactory results. However, even common surgical emergencies in transplanted patients are associated with considerable morbidity and mortality. As a general rule, surgeons treating transplanted patients should proceed with caution, apply evidence-based protocols, and expect the unexpected.

## Abbreviations

CT: Computed tomography
EAS: Emergency abdominal surgery
